# Supplementary material for: A set of multi-entry identification keys to African frugivorous flies (Diptera, Tephritidae)
Source: Zookeys. 2014 Jul 24;(428):97–108. doi: 10.3897/zookeys.428.7366 (PMC4143993; doi:10.3897/zookeys.428.7366)
Supplement: Supplementary material 6 — Key to Ceratitis [file zookeys-428-097-s006.zip › SF6_ZooKeys_key to Ceratitis/key/SF6_ZooKeys_key to Ceratitis/Media/Html/Ceratitis hamata.htm]

Ceratitis (Pardalaspis) hamata sp


***Ceratitis*** **(*Pardalaspis*) *hamata*
De Meyer**

*Ceratitis* (*Pardalaspis*)
*hamata* De Meyer, 1996 : 21.

Body
length: 6.57 (6.0-7.0) mm; wing length: 6.43 (6.0-7.0) mm.

Male

Head.
Antennal segments orange. Third antennal segment twice as long as second
segment. Arista basal part orange coloured, otherwise dark. Frons yellowish in
ground color (in specimens from Yangambi more orange), silvery over entire
length; with dispersed short hairs, at most slightly darker than frons. Ocellar
triangle dark. Face orange to orange-red; no median band. Occiput moderately
swollen below, pale whitish. Chaetotaxy normal for subgenus.

Thorax.
Ground colour of mesonotum greyish with orange tinge; also with three poorly
defined darker stripes, usually interrupted; sometimes with darker spots along
prescutellar acrostichal setae. Postpronotum slightly paler than mesonotum, no
orange tinge. Chaetotaxy normal for subgenus. Mesonotum with pale pilosity.
Anepisternum along upper margin with darker hairs, otherwise pale pilosity. Two
anepisternal bristles.  Scutellum yellow with apical markings black; basally
with two dark spots, these variable and not always distinct.

Legs
dark yellow to orange brown; setation typical of subgenus. Setae dark, front
femur with posterior row dark.

Wings
with brownish bands, yellow markings strongly reduced. Banding, setation and
venation normal for subgenus. Marginal band continuous; discal band joined with
marginal band; cross-vein r-m before middle of discal cell; vein R_1_
ending opposite cross-vein r-m.

Abdomen.
greyish, with clearly defined spots. Pattern of spots and setation normal for
subgenus.

Female

As
male except for the following characters. Third antennal segment two to three
times as long as second. Frons darker, more orange (as in males of Yangambi, cf
above); silvery shine less conspicuous than in male, along setal sockets
however pronounced; hairs distinctly darker than frons. Face wholly yellow.
Vein R_1_ ending before or opposite cross-vein r-m.  Oviscape shorter
than abdominal terga 3-6, orange in colour.   

(Description
after De Meyer, 1996)
